# Proton-activated chloride channel increases endplate porosity and pain in a mouse spine degeneration model

**DOI:** 10.1172/JCI168155

**Published:** 2024-08-28

**Authors:** Peng Xue, Weixin Zhang, Mengxi Shen, Junhua Yang, Jiachen Chu, Shenyu Wang, Mei Wan, Junying Zheng, Zhaozhu Qiu, Xu Cao

**Affiliations:** 1 Center for Musculoskeletal Research, Department of Orthopedic Surgery,; 2 Department of Physiology,; 3 Department of Biomedical Engineering, and; 4 Solomon H. Snyder Department of Neuroscience, Johns Hopkins University School of Medicine, Baltimore, Maryland, USA.

**Keywords:** Bone biology, Cell biology, Bone disease

## Abstract

Chronic low back pain (LBP) can severely affect daily physical activity. Aberrant osteoclast-mediated resorption leads to porous endplates, which allow the sensory innervation that causes LBP. Here, we report that expression of the proton-activated chloride (PAC) channel was induced during osteoclast differentiation in the porous endplates via a RANKL/NFATc1 signaling pathway. Extracellular acidosis evoked robust PAC currents in osteoclasts. An acidic environment of porous endplates and elevated PAC activation–enhanced osteoclast fusion provoked LBP. Furthermore, we found that genetic knockout of the PAC gene *Pacc1* significantly reduced endplate porosity and spinal pain in a mouse LBP model, but it did not affect bone development or homeostasis of bone mass in adult mice. Moreover, both the osteoclast bone-resorptive compartment environment and PAC traffic from the plasma membrane to endosomes to form an intracellular organelle Cl channel had a low pH of approximately 5.0. The low pH environment activated the PAC channel to increase sialyltransferase *St3gal1* expression and sialylation of TLR2 in the initiation of osteoclast fusion. Aberrant osteoclast-mediated resorption is also found in most skeletal disorders, including osteoarthritis, ankylosing spondylitis, rheumatoid arthritis, heterotopic ossification, and enthesopathy. Thus, elevated *Pacc1* expression and PAC activity could be a potential therapeutic target for the treatment of LBP and osteoclast-associated pain.

## Abstract 2

Pathological environment of porous endplates elevates proton-activated chloride channel activation in osteoclast and provokes low back pain

## Introduction

Skeletal disorders including osteoarthritis and spine degeneration are often associated with pain. Pain is a major reason people seek medical attention. Chronic low back pain (LBP) profoundly affects quality of life and daily physical activity, especially in the older population, and thus it is a key risk factor for a future decline in health (1–3). Most LBP is nonspecific with no apparent pathoanatomical cause (4). Therefore, understanding the source of LBP and its underlying mechanism is essential for its therapy. The cartilaginous endplate is composed of a thin layer of hyaline cartilage positioned between the vertebral endplate, the coronal surface of each vertebra, and the nucleus pulposus, which is the inner core of the vertebral disc that acts as the shock absorber for each spinal unit. Our previous studies have demonstrated that aberrant osteoclast-mediated resorption of calcified cartilaginous endplates during spine degeneration generates a porous structure, and osteoclasts in porous endplates secrete netrin-1, which allows for sensory innervation of the spinal unit, thus leading to LBP (5–7). However, it is still unclear how osteoclast activity becomes aberrant during spinal degeneration.

Pain in skeletal disorders is often associated with aberrant osteoclast resorption in very low pH environments (8, 9). Our recent study revealed that the proton-activated chloride (PAC) channel is a proton-activated Cl^−^ channel (encoded by *Pacc1*, also known as TMEM206) that is opened by acidic pH. The channel is responsive to pathological acidic pH in ischemic brain injury and acid-induced neuronal cell death in mice (10). PAC represents a completely new ion channel family that has no obvious sequence homology to other membrane proteins, but it is highly conserved in vertebrates (11). There are two-transmembrane (TM) helices in PAC, like the acid-sensing ion channel (ASIC) and the epithelial sodium channel (ENaC) (12). The PAC structure study showed that the protein exists in 2 states: namely, a high-pH, resting closed state and a low-pH, proton-bound nonconducting state. The PAC channel undergoes striking conformational changes when the pH drops from 8 to 4, leading to an opening of the channel and the conduction of anions across cellular membranes, thereby inducing diseases associated with tissue acidosis (acid-induced cell death). Interestingly, PAC also localizes in intracellular organelles (endosomes and macropinosomes) and regulates their pH and volume homeostasis (13). The aberrant osteoclast bone remodeling in some of the major bone diseases such as osteoarthritis, rheumatoid arthritis, and LBP are probably associated with low pH, therefore, we set out to investigate the function of the PAC channel in osteoclast differentiation and resorption in LBP.

Osteoclasts are formed from macrophages and monocytes that, in response to NF-κB ligand (RANKL) signaling, become committed to a tartrate-resistant acid phosphatase–positive (TRAP^+^) osteoclast lineage (14, 15). TRAP^+^ mononuclear cells first attach to the bone surface and then undergo fusion to form multinucleated osteoclasts (16). Osteoclasts are polarized to form ruffle membranes with abundant ATPase proton pump activity at the side attached to the bone (17). Chloride channels such as CIC-7 are important in the cell membrane and intracellular organelles (18). In osteoclasts, CIC-7 is predominantly localized to the ruffled border, a specialized membrane domain crucial for acidifying the resorption lacuna. This acidification process supports ATPase proton pump activity, thereby enabling bone resorption. CIC-7 is also expressed at the membrane of lysosomes and endosomes (19). At both the lysosome/endosome membrane and the ruffled border, CIC-7 functions in the ionic homeostasis and maintains the pH (20, 21). The compartment between the ruffled membrane and the bone surface within the osteoclast sealing zone is acidified by secretion of protons, leading to dissolution of the bone matrix material (22, 23). Aberrant osteoclast-mediated bone resorption and the secretion of netrin-1 are associated with many major skeletal disorders, including LBP, osteoarthritis, heterotopic ossification, and ankylosing spondylitis, among others (5, 24–28). PAC is activated in low-pH environments with implications for bone diseases, and it is imperative to understand its function in osteoclast differentiation and resorption in conjunction with CIC-7.

In this study, we investigated the potential role of PAC in osteoclast function in the porous endplates in spine degeneration–associated LBP. We found that PAC expressed on both the cell membrane and in the cytoplasm of osteoclasts and its open conformation only occurred under an acidic microenvironment. Knockout of *Pacc1* markedly reduced osteoclast fusion and endplate porosity, as well as relieved LBP in a mouse model of spine degeneration, but the genetic deletion did not have any effect on bone development or bone homeostasis in adult mice. Together, our results show that *Pacc1*-encoded Cl^−^ channel activity was induced during acidosis, leading to aberrant osteoclast-mediated resorption that generated endplate porosity and resulted in LBP. As depletion of PAC activity prevented the development of LBP during spine degeneration with no negative effect on bone modeling or remodeling, this represents a potential therapeutic target for LBP.

## Results

### Knockout of Pacc1 significantly reduces spinal pain and endplate porosity in a mouse model of spine degeneration.

**Figure 1:**
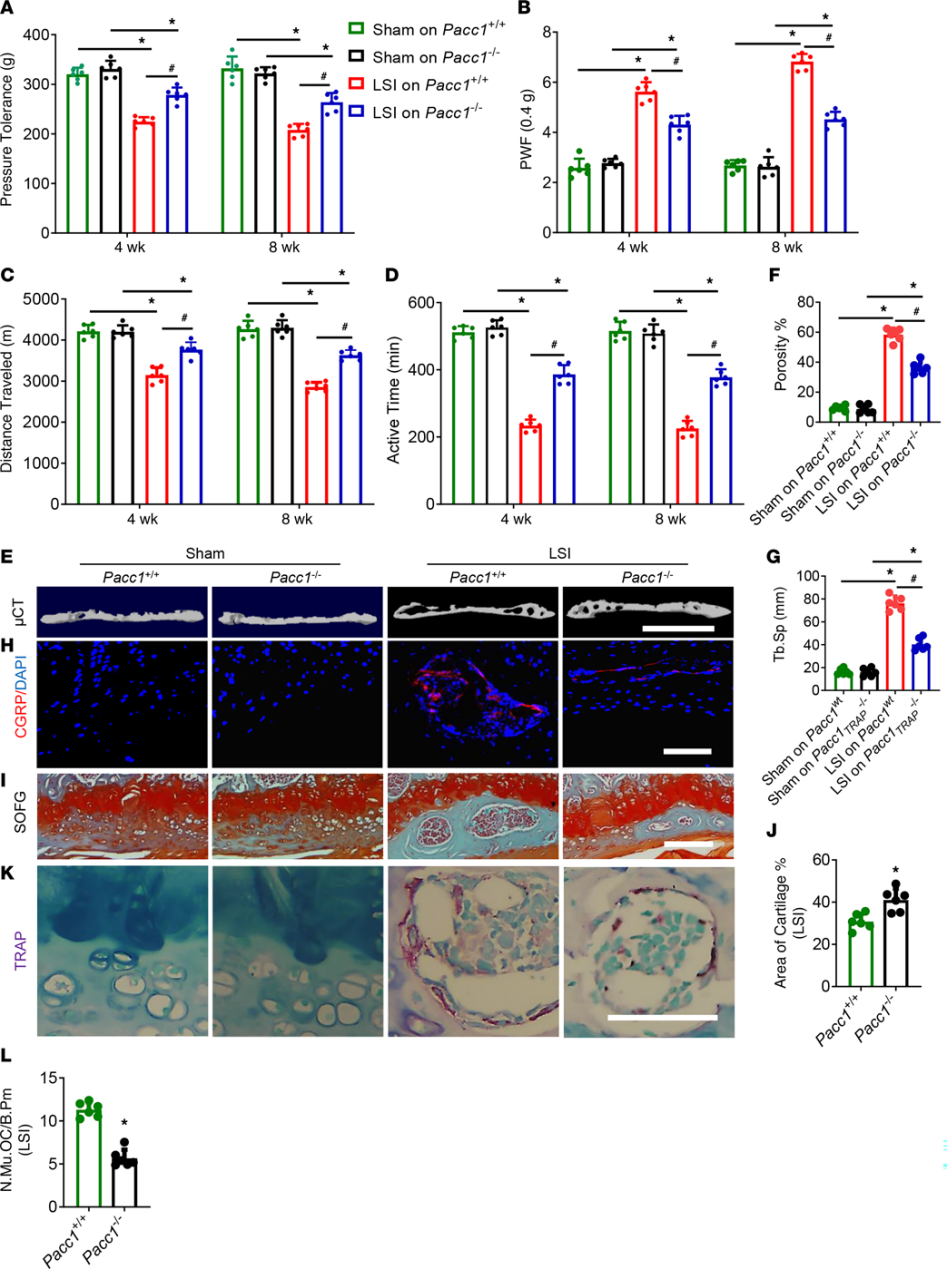
Knockout of *Pacc1* significantly reduces spinal pain and endplate porosity in a mouse model of spine degeneration. (**A**) Pressure tolerance of the lumbar spine as assessed by the force threshold needed to induce vocalization by a force gauge 4 and 8 weeks after LSI surgery. (**B**) Hind PWF in response to mechanical stimulation (von Frey, 0.4 g) 4 and 8 weeks after LSI surgery. (**C** and **D**) Spontaneous activity analysis, including distance traveled (**C**) and active time (**D**) on the wheel per 24-hour period. (**E**) Representative 3D, high-resolution μCT images of the L4–L5 caudal endplates (coronal view) 8 weeks after LSI surgery (*n* = 6 per group). Scale bar: 1 mm. (**F** and **G**) Quantitative analysis of total porosity (**F**) and trabecular separation (Tb.Sp) (**G**) of the L4–L5 caudal endplates as determined by μCT. (**H**) Representative images of CGRP (red) and DAPI (blue) immunofluorescence staining of nerve fibers in the endplates 8 weeks after LSI surgery. Scale bar: 50 μm. (**I**) Representative images of SOFG staining of coronal L4–L5 caudal endplate sections 8 weeks after LSI surgery. Scale bar: 50 μm. (**J**) Quantitative analysis of the area of cartilage in the endplates of *Pacc1^+/+^* and *Pacc1^–/–^* mice 8 weeks after LSI surgery. (**K**) Representative images of coronal L4–L5 caudal endplate sections stained for TRAP 8 weeks after LSI surgery. Scale bar: 50 μm. (**L**) Quantitative analysis of the number of TRAP^+^ multinuclear cells in endplates. N.Mu., number of multinuclear osteoclasts; OC, osteoclast; B.Pm, bone perimeter. (**A**–**D**, **F**, and **G**) *P < 0.05 compared with the sham group; ^#^*P* < 0.05 compared with *Pacc1^+/+^* mice after LSI surgery (*n* = 6 per group). (**J** and **L**) *P < 0.05 compared with the *Pacc1^+/+^* group, by 1-way ANOVA (**A**, **D**, **F**, and **F**) and 2-tailed *t* test (**J** and **L**) (*n* = 6 per group). Data are presented as the mean ± SD.

During spine degeneration, osteoclast activity is stimulated, leading to porosity of the endplates, and netrin-1 secreted by osteoclasts induces calcitonin gene–related peptide–positive (CGRP^+^) nerve innervation and thus causes LBP. To examine the potential role of PAC in osteoclast-mediated resorption of the endplate and the generation of its porosity, we used mice with genetic deletion of *Pacc1* (10). We then surgically manipulated the *Pacc1^–/–^* mice and their WT littermates to generate a mouse model of lumbar spine instability (LSI) as a form of spine degeneration. Through pain behavior tests, we found that pressure tolerance was significantly lower in the WT LSI mice at 4 and 8 weeks after LSI induction compared with sham-treated WT mice, whereas the degree of change in pressure tolerance in the *Pacc1^–/–^* LSI mice was significantly less than in the WT LSI mice but still lower than that seen in the sham-operated mutant mice (Figure 1A). These results suggest that *Pacc1*-encoded activity is associated with endplate porosity–induced LBP. Furthermore, by measuring the paw withdrawal frequency (PWF) in von Frey tests to evaluate mechanical pain hypersensitivity, we found that mechanical hyperalgesia was significantly lower at 4 and 8 week after LSI surgery in *Pacc1^–/–^* LSI mice relative to their WT LSI littermates (Figure 1B). We also conducted a spontaneous activity behavior test. The distance traveled and active time per 24-hour period were significantly greater in *Pacc1^–/–^* LSI mice relative to their WT LSI littermates (Figure 1, C and D).

Next, we examined the sclerosis endplates by micro-CT (μCT) and found that endplate porosity was significantly lower in *Pacc1^–/–^* LSI mice relative to WT LSI mice 8 weeks after LSI induction (Figure 1, E–G). By immunostaining for CGRP as a readout of innervation, we found that *Pacc1^–/–^* LSI mice had less sensory nerve innervation in the porous endplates than did WT LSI mice (Figure 1H). We then stained L4–L5 caudal endplates with Safranin O and fast green (SOFG) staining and found a larger cartilage area and smaller porosity proportion in the endplate of the *Pacc1^–/–^* LSI mice relative to WT LSI mice 8 weeks after surgery (Figure 1, I and J). Strikingly, by TRAP staining we found that the number of large, multinuclear osteoclasts was lower in the *Pacc1^–/–^* LSI mice relative to WT LSI mice 8 weeks after surgery (Figure 1, K and L), suggesting that *Pacc1* expression promoted aberrant osteoclast fusion and function. Together, these results indicate that PAC plays a role in pathological osteoclast bone resorption during the generation of endplate porosity and LBP during LSI.

**Figure 2:**
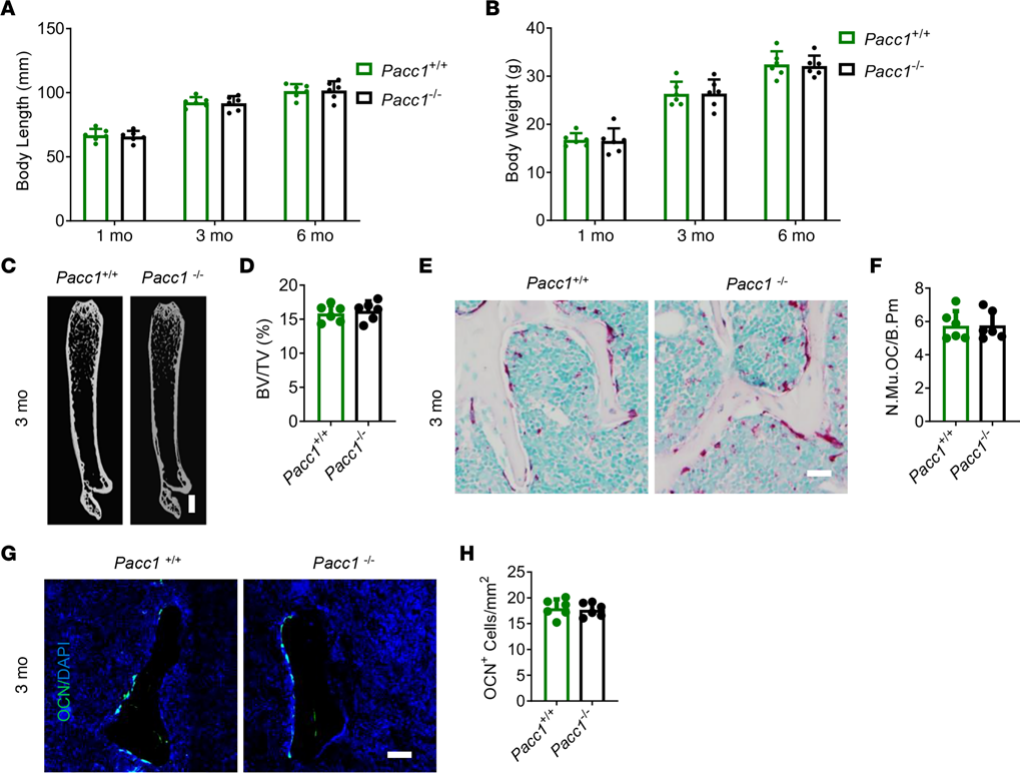
Knockout of the *Pacc1* channel does not influence bone development or femur bone mass in adult mice. (**A**) Body length of *Pacc1^+/+^* and *Pacc1^–/–^* mice (*n* ≥5). (**B**) Body weight of *Pacc1^+/+^* and *Pacc1^–/–^* mice (*n* ≥5). (**C**) Representative μCT images of femurs from 3-month-old male *Pacc1^+/+^* and *Pacc1^–/–^* mice. Scale bar: 1 mm. (**D**) Quantitative analysis of μCT result for BV/TV in femurs from 3-month-old male *Pacc1^+/+^* and *Pacc1^–/–^* mice (*n* = 6). (**E**) Representative images of TRAP staining of coronal femur sections from 3-month-old male *Pacc1^+/+^* and *Pacc1^–/–^* mice. Scale bar: 50 μm. (**F**) Quantitative analysis of the number of TRAP^+^ multinuclear cells in femurs (*n* = 6). (**G**) Representative images of immunofluorescent analysis of OCN staining and DAPI (blue) staining of nuclei for coronal femur sections from 3-month-old male *Pacc1^+/+^* and *Pacc1^–/–^* mice. Scale bar: 50 μm. (**H**) Quantitative analysis of the number of OCN^+^ cells in femurs (*n* = 6). Data are presented as the mean ± SD.

We also examined whether PAC expression plays a physiological role in normal osteoclast bone resorption, bone development, and bone remodeling. First, we investigated the effect of PAC on skeletal development and adult bone homeostasis. We examined the body length and weight at 1, 3, and 6 months of age and found that *Pacc1^–/–^* mice showed no difference compared with their WT littermates (Figure 2, A and B). Notably, we measured various bone parameters in the mice during development and adulthood by μCT at 1, 3, and 6 months of age. We found that an important bone parameter, bone volume, in *Pacc1^–/–^* mice was not different than that of WT *Pacc1^+/+^* mice at 3 months of age (Figure 2, C and D). Furthermore, by TRAP staining of femur sections, we found that osteoclast numbers did not change in *Pacc1^–/–^* mice relative to those for WT *Pacc1^+/+^* mice at 3 months of age (Figure 2, E and F). Furthermore, using osteocalcin (OCN) staining, we found no difference in bone formation between *Pacc1^–/–^* and WT mice at 3 months of age (Figure 2, G and H). Taken together, these results indicate that PAC expression did not play a critical role in osteoclast bone resorption during bone development or in bone homeostasis under physiological conditions.

### PAC expression is induced during osteoclast differentiation.

**Figure 3:**
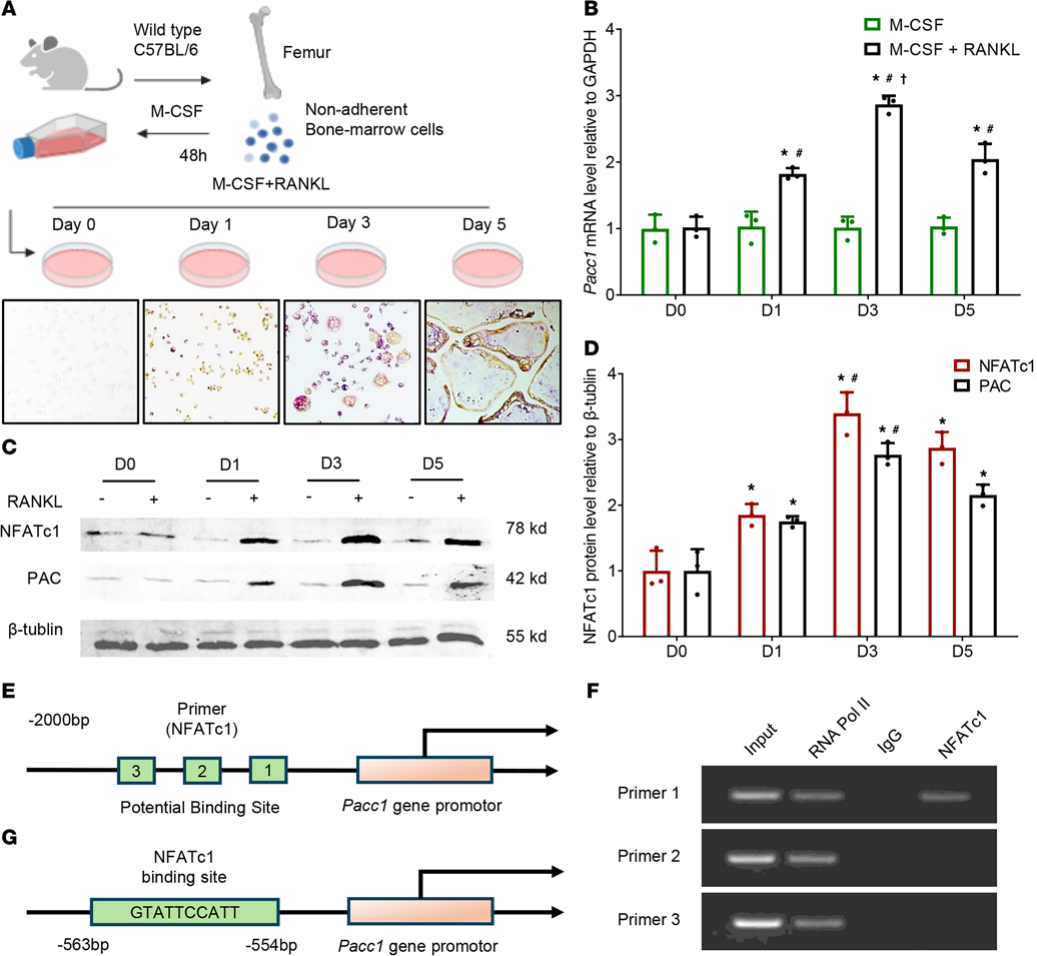
PAC expression is induced during osteoclast differentiation. (**A**) Schematic diagram illustrating the preparation of TRAP^+^ osteoclast precursors and their maturation process. (**B**) Relative mRNA expression levels of *Pacc1* in the cells cultured in the medium with or without RANKL at day 0 (D0), day 1 (D1), day 3 (D3), and day 5 (D5) (*n* = 3). (**C** and **D**) Protein levels of NFATc1 and PAC at 0, 1, 3, and 5 days were analyzed by Western blotting (*n* = 3). (**E**) Diagram of potential NFATc1 binding sites on the *Pacc1* promoter in osteoclast precursors. (**F**) ChIP analysis of NFATc1 on the *Pacc1* promoter. (**G**) Diagram of the *Pacc1* promoter with an NFATc1 binding site. (**B**) *P < 0.05 compared with day 0; ^#^*P* < 0.05 compared with the M-CSF group (without RANKL); ^†^*P* < 0.05 compared with day 1. (**D**) *P < 0.05 compared with day 0; ^#^*P* < 0.05 compared with day 1. Significance was determined by 2-way ANOVA (**B** and **D**). Data are presented as the mean ± SD.

To determine whether PAC expression is induced during osteoclast differentiation and resorption, whole bone marrow cells were isolated from C57BL/6 mouse hind limbs and cultured with macrophage colony-stimulating factor (M-CSF) (50 ng/mL) for 2 days to induce the growth of bone marrow macrophages (BMMs). BMMs were then treated with M-CSF (30 ng/mL) and RANKL (100 ng/mL) for 5 days to induce osteoclast differentiation, as well as their fusion into mature osteoclasts (mOCs) (Figure 3A). *Pacc1* mRNA expression was significantly induced by RANKL stimulation relative to the M-CSF group at 1, 3, and 5 days and peaked at day 3, as measured by reverse transcription PCR (RT-PCR) (Figure 3B). To confirm expression of the *Pacc1*-encoded channel protein during osteoclast differentiation, we treated BMMs with RANKL and harvested them at days 0, 1, 3, and 5 for Western blot analysis of PAC expression. Consistent with the pattern of mRNA expression, we found that PAC expression was higher in the RANKL-treated cells than in BMMs treated with M-CSF alone, with peak expression at day 3 (Figure 3, C and D). We next measured the expression of NFATc1, a downstream transcription factor induced by RANKL signaling, and found that its expression was greater with RANKL treatment in a time-dependent manner that matched that of PAC, suggesting that NFATc1 transcriptionally regulated *Pacc1* expression. We found 3 potential NFATc1-binding sites in the *Pacc1* promoter (Figure 3E), and by a ChIP assay, we demonstrated that RANKL induced specific binding of NFATc1 to the most proximal NFATc1-binding site of the *Pacc1* promoter to activate its expression in osteoclasts (Figure 3, F and G). Thus, PAC expression was induced during osteoclast maturation and bone resorption.

### Extracellular acidosis evokes robust PAC currents in osteoclasts.

**Figure 4:**
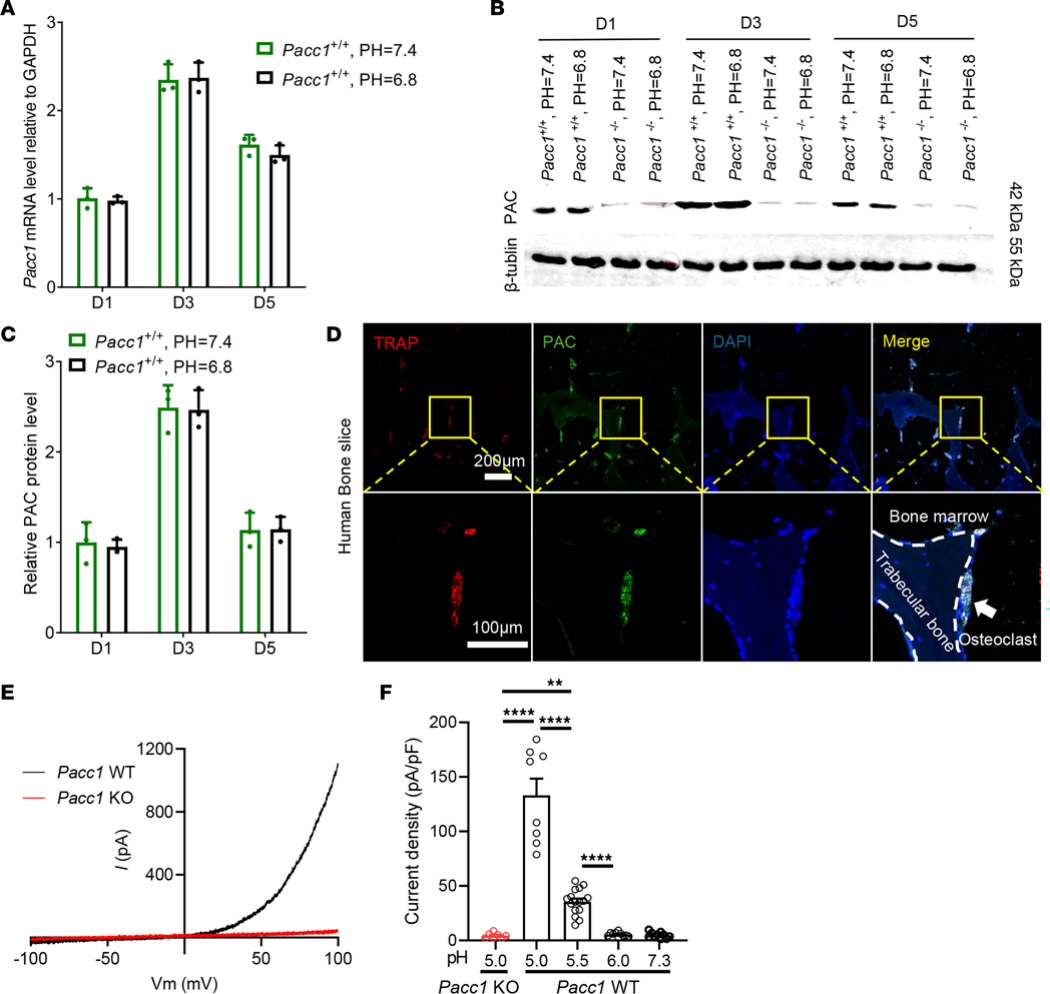
Extracellular acidosis evokes the *I*_Cl,__H_ current in the cell membrane of osteoclasts by activating PAC. (**A**) Relative mRNA expression levels of *Pacc1* in the cells isolated from *Pacc1^+/+^* mice; cells were cultured in neutral or acidic medium at 1, 3, and 5 days (*n* = 3). (**B** and **C**) Protein levels of PAC in the cells isolated from *Pacc1^+/+^* or *Pacc1^–/–^* mice cultured in neural or acidic medium at 1, 3, and 5 days (*n* = 3). (**D**) Representative images of coimmunofluorescence staining for PAC (green) and TRAP (red) in human bone section. DAPI (blue). Scale bars: 200 μm and 100 μm. (**E**) PAC currents monitored by a voltage-ramp protocol at pH 5.5 in the cells isolated from *Pacc1^+/+^* and *Pacc1^–/–^* mice; cells were cultured in osteoclastic medium for 3 days. (**F**) PAC-mediated current densities measured at +100 mV in cells isolated from *Pacc1^–/–^* (red) and *Pacc1^+/+^* (black) mice; cells were cultured in osteoclastic medium for 3 days, at pH 5.0, 5.5, 6.0, and 7.3 at 37°C (*n* ≥7). (**E** and **F**) **P < 0.01 and ****P < 0.001 compared with cells isolated from *Pacc1^+/+^* mice. Significance was determined by 2-way ANOVA (**A** and **C**) and 1-way ANOVA (**F**). Data are presented as the mean ± SD.

To examine the functional activity of PAC, BMMs were isolated from WT and *Pacc1^–/–^* mice and cultured with neutral (pH = 7.4) or acidic (pH = 6.8) medium containing M-CSF and RANKL. By RT-PCR and Western blot analysis, we found that extracellular acidosis did not affect *Pacc1* or PAC expression at 1, 3, and 5 days of osteoclast differentiation (Figure 4, A–C). Furthermore, coimmunostaining of PAC with TRAP on human bone sections demonstrated that PAC was expressed on the cellular membrane and intracellular organelles of osteoclasts and well colocalized with TRAP staining at the bone surface (Figure 4D). Indeed, using whole-cell patch clamping, we found that extracellular acidosis evoked the PAC currents in *Pacc1^WT^* preosteoclasts at day 3 after RANKL treatment, while they were absent in *Pacc*1*^–/–^* cells (Figure 4, E and F). These results demonstrate that PAC was functionally expressed in osteoclast lineage cells and that the channel was activated under acidic conditions. The results suggest that the function of PAC, unlike the chloride transporter CIC-7, is not specific to osteoclast bone resorption, but likely regulates osteoclast fusion in a low-pH environment.

### Knockout of Pacc1 in TRAP^+^ cells reduces spinal pain and endplate porosity in the LSI model.

**Figure 5:**
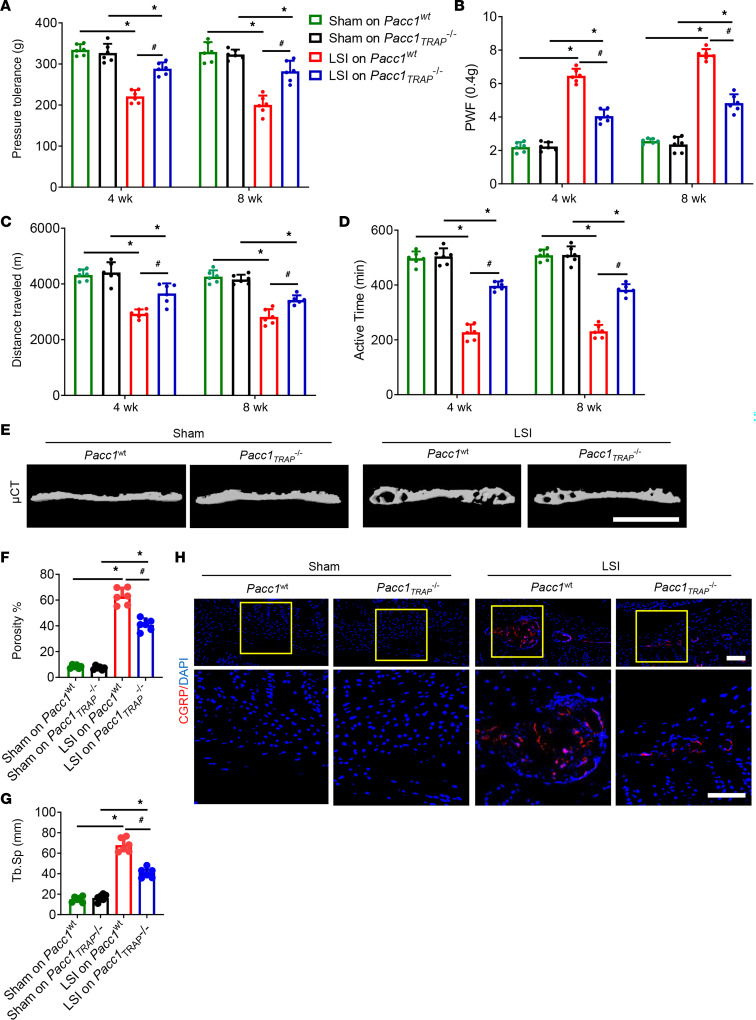
Knockout of the PAC channel in TRAP^+^ cells reduces spinal pain and endplate porosity in the LSI model. (**A**) Pressure tolerance of the lumbar spine as assessed by the force threshold needed to induce vocalization by a force gauge 4 and 8 weeks after LSI surgery. (**B**) Hind PWF in response to mechanical stimulation (von Frey, 0.4 g) 4 and 8 weeks after LSI surgery. (**C** and **D**) Spontaneous activity analysis, including distance traveled (**C**) and active time (**D**) on the wheel per 24-hour period. (**E**) Representative 3D, high-resolution μCT images of the L4–L5 caudal endplates (coronal view) 8 weeks after LSI surgery. Scale bar: 1 mm. (**F** and **G**) Quantitative analysis of total porosity (**F**) and trabecular separation (**G**) of the L4–L5 caudal endplates as determined by μCT. (**H**) Representative images of immunofluorescence analysis of CGRP (red) and DAPI (blue) staining of nerve fibers in endplates 8 weeks after LSI surgery. Scale bars: 50 μm. (**A**–**D**, **F**, and **G**) *P < 0.05 compared with the sham group; ^#^*P* < 0.05 compared with *Pacc1^WT^* mice after LSI surgery (*n* = 6 per group). Significance was determined by 1-way ANOVA. Data are presented as the mean ± SD.

To examine how PAC expression in osteoclasts induces LBP, we crossed floxed *Pacc1* mice (*Pacc1^fl/fl^*) with TRAP-Cre mice to generate conditional *Pacc1_TRAP_^–/–^* mice. We then conducted a series of pain behavior tests with *Pacc1_TRAP_^–/–^* mice and found that pressure tolerance was significantly greater in *Pacc1_TRAP_^–/–^* LSI mice relative to that of *Pacc1^WT^* LSI mice at both 4 and 8 weeks after the operation (Figure 5A). Moreover, mechanical hyperalgesia, as measured by von Frey tests, was lower in *Pacc1_TRAP_^–/–^* LSI mice at 4 and 8 weeks after the operation compared with *Pacc1^WT^* LSI mice (Figure 5B). In addition, spontaneous activity, which included distance traveled and activity time over a 24-hour period, was significantly greater for *Pacc1_TRAP_^–/–^* LSI mice relative to *Pacc1^WT^* LSI mice at both 4 and 8 weeks after surgery (Figure 5, C and D). As expected, μCT scanning revealed that there was less endplate porosity and trabecular separation in *Pacc1_TRAP_^–/–^* LSI mice relative to *Pacc1^WT^* LSI mice at 8 weeks after surgery (Figure 5, E–G). And by immunostaining for CGRP, we found less sensory nerve innervation in the porous endplates in *Pacc1_TRAP_^–/–^* LSI mice compared with *Pacc1^WT^* LSI mice (Figure 5H). Therefore, PAC expression enhanced osteoclast resorptive activity to generate porous endplates, leading to pain hypersensitivity in a mouse model of spine degeneration.

### PAC-mediated I_Cl,H_ current activity in response to extracellular acidosis enhances osteoclast fusion.

**Figure 6:**
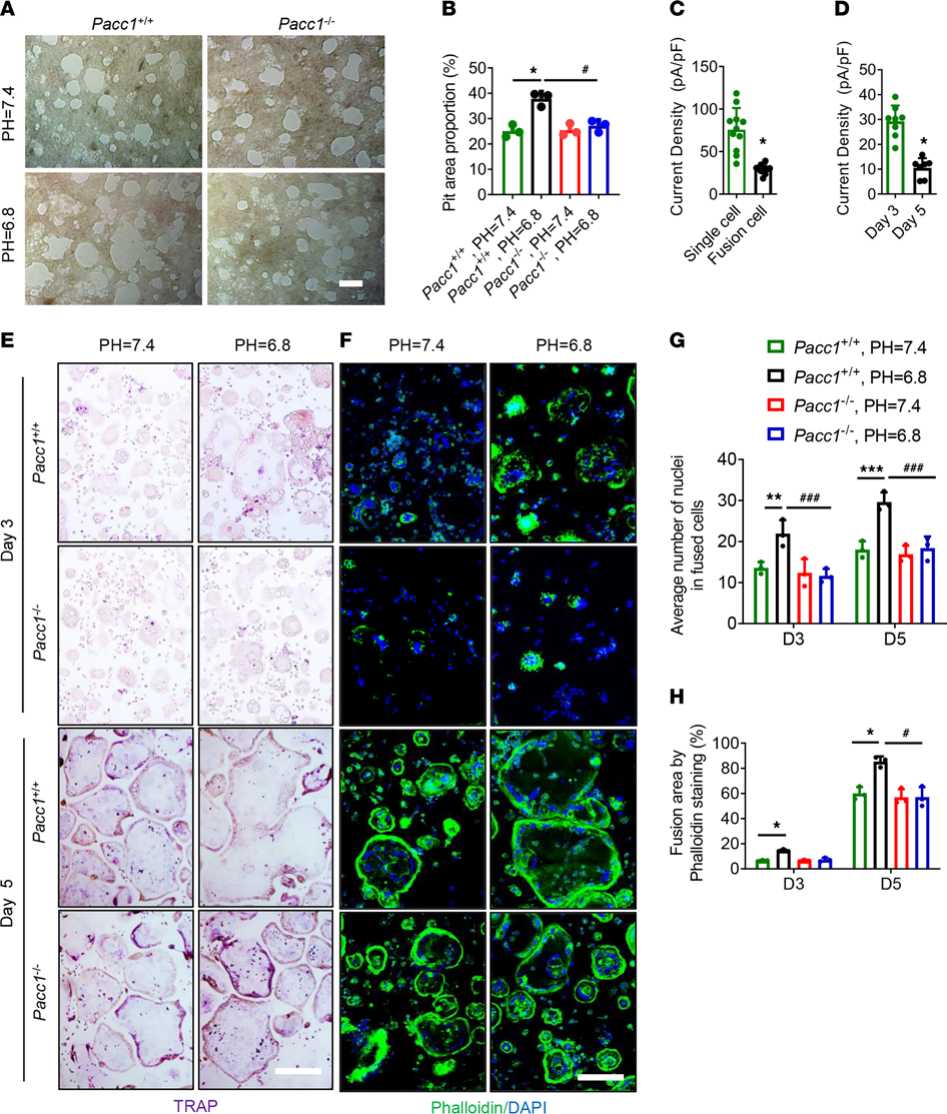
PAC-mediated *I*_Cl,__H_ current activity in response to extracellular acidosis enhances osteoclast fusion and resorption. (**A** and **B**) Resorption pits of the cells isolated from *Pacc1^+/+^* and *Pacc1^–/–^* mice; cells were cultured in neural or acidic medium at day 7 (*n* = 3). Scale bar: 50 μm. (**C**) PAC-mediated currents in single cells (osteoclast precursors) or fused cells (mature osteoclasts) cultured in osteoclastic medium for 3 days (*n* ≥7). (**D**) PAC-mediated currents in the cells cultured in osteoclastic medium for 3 or 5 days (*n* ≥7). (**E** and **G**) TRAP staining of cells isolated from *Pacc1^+/+^* and *Pacc1^–/–^* mice; cells were cultured in neural or acidic medium at day 3 and day 5 (*n* = 3). Scale bar: 50 μm. (**F** and **H**) Phalloidin staining for cells isolated from *Pacc1^+/+^* or *Pacc1^–/–^* mice cultured in neural or acidic medium at 3 and 5 days (*n* = 3). Scale bar: 50 μm. (**B**, **G**, and **H**) *P < 0.05 compared with cells isolated from *Pacc1^+/+^* mice cultured in the neutral medium; ^#^*P* < 0.05 compared with cells isolated from *Pacc1^+/+^* mice cultured in the acidic medium. (**C**) *P < 0.05 compared with the single cell. (**D**) *P < 0.05 compared with day 3. Significance was determined by 1-way ANOVA (A, B, G, and H) and 2-tailed *t* test (**C** and **D**). Data are presented as the mean ± SD.

Next, we investigated PAC functional activity during osteoclast-mediated bone resorption. Osteoclasts prepared from either *Pacc1^–/–^* or *Pacc1^WT^* mice were cultured on bone slices to examine their bone-resorptive activity, which is referred to as a pit assay. The bone-resorptive areas were significantly larger underneath osteoclasts from *Pacc1^WT^* mice in pH 6.8 medium relative to pH 7.4 (Figure 6, A and B), an effect that was blunted among osteoclasts from *Pacc1^–/–^* mice in pH 6.8 medium, indicating that PAC expression enhanced the bone resorption ability of osteoclasts in a mildly low-pH environment. We recorded a higher activated proton Cl channel current (*I*_Cl,H_ current) on the membrane of TRAP^+^ mononuclear cells than of multinuclear cells at day 3 after RANKL treatment (Figure 6C). The PAC-mediated *I*_Cl,H_ current of multinuclear cells was significantly lower at day 5 relative to day 3 after RANKL treatment (Figure 6D), indicating that PAC primarily regulated TRAP^+^ mononuclear cells prior to their osteoclast fusion. Moreover, by TRAP staining we found that extracellular acidosis accelerated osteoclast fusion in BMMs isolated from *Pacc1^+/+^* mice in pH 6.8 medium at days 3 and 5 after RANKL treatment (Figure 6, E and G, and Supplemental Figure 1A; supplemental material available online with this article; https://doi.org/10.1172/JCI168155DS1). The results were further confirmed by phalloidin staining (Figure 6, F and H, and Supplemental Figure 1A), which showed that osteoclast fusion in response to an acidic medium was blunted in BMMs from *Pacc1^–/–^* mice. Taken together, our data indicate that PAC was required for osteoclast fusion at low pH.

### PAC is essential for expression of the sialyltransferase St3gal1 in osteoclast fusion through its induction of sialylation of TLR2.

**Figure 7:**
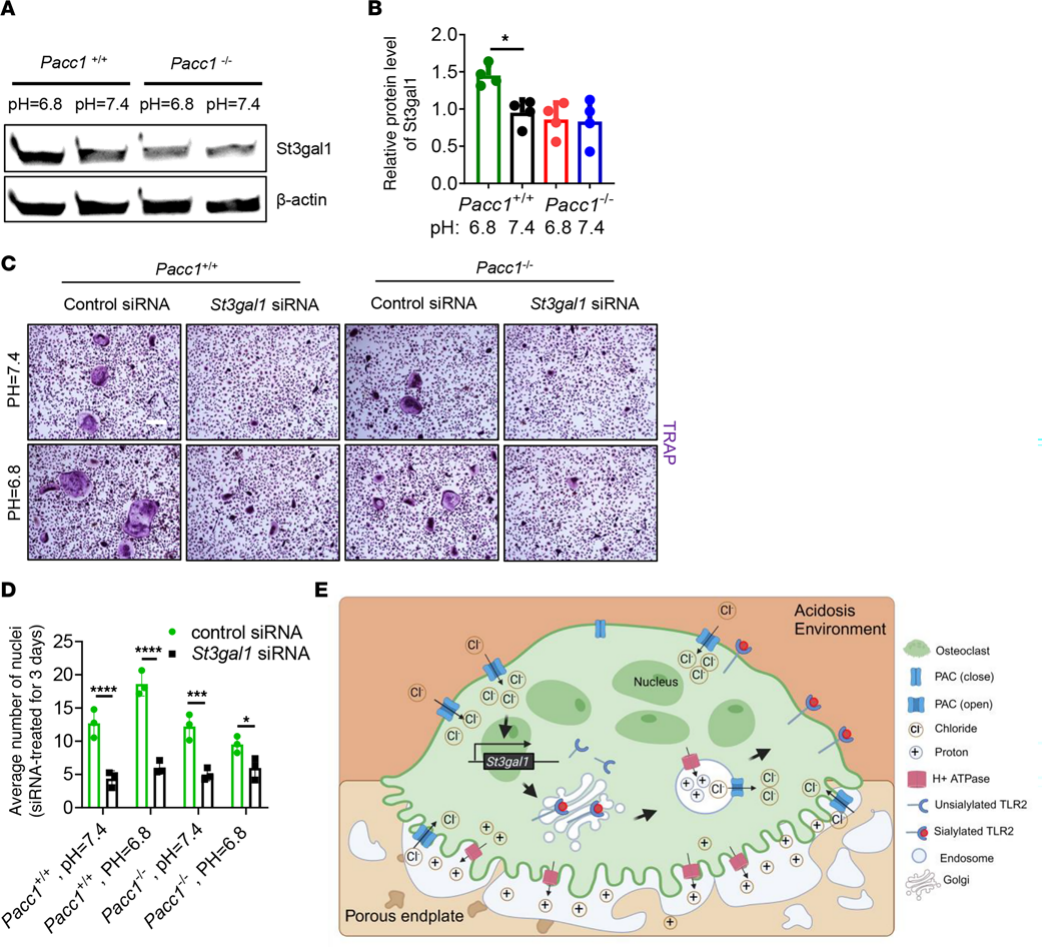
PAC mediates osteoclast fusion through sialyltransferase St3gal1-induced sialylation of TLR2. (**A**) Representative image and quantitative analysis of Western blot for St3gal1 protein expression relative to β-actin in BMMs isolated from *Pacc1^+/+^* and *Pacc1^–/–^* mice, at pH 6.8 or pH 7.4. (**B**) Statistical analysis of St3gal1 protein expression in each group, relative to the *Pacc1^+/+^* pH 7.4 group (*n* = 4). (**C** and **D**) TRAP staining of cells isolated from *Pacc1^+/+^* and *Pacc1^–/–^* mice; cells were cultured in neural or acidic medium for 3 days with RANKL stimulation and control or *St3gal1* siRNA interference (*n* = 3). Scale bar: 50 μm. (**E**) The acidosis environment in the LSI model could acidify the intracellular pH level in osteoclasts through a synergetic function of PAC on the membrane. The translational expression of *St3gal1* is regulated by PAC under the low-pH condition. PAC on the membrane of the endosome is responsible for maintaining the posttranslational sialylation of TLR2, which is mediated by St3gal1 for osteoclast fusion. (**B** and **D**) *P < 0.05, ***P < 0.005, and ****P < 0.001. Significance was determined by 1-way ANOVA (**B**) and 2-way ANOVA (**D**). Data are presented as the mean ± SD.

We have reported that sialyltransferase, St3gal1-mediated sialylation of TLR2 on pre-osteoclasts initiates osteoclast fusion (29, 30). To investigate whether PAC regulates osteoclast fusion through the sialylation of TLR2, BMMs isolated from *Pacc1^+/+^* and *Pacc1^–/–^*mice were treated with M-CSF and RANKL for 3 days under acidic and physiological pH conditions, as we described above. Total protein was harvested for Western blot analysis. We found that the expression of St3gal1 was minimally expressed at pH 7.4 but significantly increased at pH 6.8. Importantly, St3gal1 expression was significantly decreased in *Pacc1^–/–^* cells in both acidic and physiological pH media (Figure 7, A and B), suggesting that activation of PAC at low pH promoted the expression of St3gal1 to initiate preosteoclast fusion through St3gal1-mediated TLR2 sialylation. To determine whether PAC is essential for *St3gal1* expression to initiate osteoclast fusion, we knocked down *St3gal1* in *Pacc1^+/+^* and *Pacc1^–/–^* BMMs using an siRNA against *St3gal1* (150809, Thermo Fisher Scientific), and the result showed that the number of fused cells significantly decreased in *St3gal1* siRNA–treated groups relative to control siRNA–treated groups under different pH conditions (Figure 7, C and D, and Supplemental Figure 1, B–E). The osteoclast fusion marker OC-STAMP was also significantly decreased in the *St3gal1-*knockdown groups in different pH environments (Supplemental Figure 1, F–I). Thus, in a low-pH microenvironment, *St3gal1* expression required PAC activity to induce sialylation of TLR2 for osteoclast fusion.

## Discussion

Endplates are cartilaginous structures connecting the vertebral body with the intervertebral disc in the spine. Endplates undergo porous sclerosis with partial ossification in patients with spine degeneration. Normally, osteoclasts do not resorb cartilage, but they can target partially calcified cartilage in the porous endplates, in which the confined environment is marked by sensory innervation, angiogenic type H vessels, and a low pH. Importantly, aberrant osteoclast resorption is found in many skeletal disorders with similar pathological environments including osteoarthritis (24, 28), ankylosing spondylitis (25), rheumatoid arthritis (31, 32), enthesopathy (33), spine degeneration (5), heterotopic ossification (27), and Paget disease (34). Osteoclast-mediated resorptive activity in the pathological environment produces excessive netrin-1, PDGF-BB, TGF-β, and IGF-1 to promote the progression of skeletal disorders and pain (35–37). In our LSI animal model, the pH in the porous endplates we measured was 6.92 ± 0.08 (Supplemental Figure 2A). In addition, the Warburg effect is known as an important mechanism in generating an acidic microenvironment through elevated expression of lactic dehydrogenase A (LDHA) (38, 39). We found that LDHA significantly accumulated in endplates of LSI mice (Supplemental Figure 2, B and C). Moreover, PAC expression is induced by NFATc1 with RANKL stimulation on the membrane and intracellular organelles of osteoclasts, as RANKL induces the commitment of macrophages to the TRAP^+^ osteoclast lineage for osteoclast fusion and maturation (29, 30). Interestingly, both the osteoclast bone-resorptive compartment environment and PAC traffic from the plasma membrane to endosomes to form an intracellular organelle Cl channel have a low pH of approximately 5.0. The low-pH environment activates the PAC channel to increase *St3gal1* expression for sialylation of TLR2 in the initiation of osteoclast fusion.

Chloride channels and transporters such as CIC-7, encoded by the *Clcn7* gene, are important in the cell membrane and intracellular organelles (18). CIC-7 is primarily localized at the ruffled border of osteoclasts. The ruffled border is a special membrane area that is important for acidification and bone resorption. CIC07 is also expressed at the membrane of lysosomes and endosomes (19). At both the lysosome and endosome membrane and the ruffled border, CIC-7 contributes to ionic homeostasis and maintains the pH (20, 21). Importantly, CIC-7 provides the chloride conductance in endosomes and lysosomes, along with proton pumping in the ruffled membrane of osteoclasts (18, 19, 21). When we compared CIC-7 expression, which was polarized in the osteoclast ruffled membrane to allow for chloride conductance to endosomes and lysosomes, along with proton pumping, we found that PAC was evenly distributed on the membrane of TRAP^+^ mononuclear cells and osteoclasts, with the primary function being to induce osteoclast fusion. PAC was activated at low pH to induce the expression of *St3gal1*, which in turn would lead to sialylation of TLR2 for the fusion of TRAP^+^ mononuclear cells (Figure 7, A and B). Knockout of *Pacc1* reduced osteoclast fusion in the endplates of LSI mice, whereas osteoclast fusion and bone resorption were not affected in normal bone. At low pH, *Pacc1^–/–^* preosteoclasts were difficult to fuse for the formation of osteoclast fusion. Thus, one of the important functions of PAC is to maintain osteoclast fusion in a low-pH environment.

We demonstrated that an acidic environment promoted *Pacc1^+/+^*, but not *Pacc1^–/–^*, osteoclast fusion, while resorption was increased by approximately 50% due to the “sudden stimulus” as opposed to continuous cultivation, as Tim Arnett described (40). Tim Arnett et al. uncovered a significant effect of extracellular protons on the osteoclast bone resorption. This study represents the initial direct evidence that low pH enhances cell-mediated bone resorption (40). Furthermore, they found that rat osteoclasts may be more sensitive to stimulation by CO_2_ acidosis than by HCO_3_^–^ acidosis (41). Arnett’s group investigated the effect of small shifts in extracellular pH on the resorptive activity of rat osteoclasts in vitro and found that very slight alterations in ambient hydrogen ion concentration can effectively “switch on” or “switch off” rat osteoclasts in vitro (42). His group also examined the effects of HCO_3_^–^ and CO_2_ acidosis on osteoclast-mediated Ca^2+^ release from 3-day cultures of neonatal mouse calvaria and found that addition of H^+^ reduced the pH from 7.12 to 7.03 and increased Ca^2+^ release by 3.8-fold and that CO_2_ acidosis was a less effective stimulator of Ca^2+^ release than HCO_3_^–^ acidosis over a similar pH range (43).

TRAP could be expressed in other cell types, such as leukocytes. According to our results, bone homeostasis remained unchanged in PAC global-knockout mice. Furthermore, TRAP conditional-knockout *Pacc1_TRAP_^–/–^* mice displayed osteoclast functional outcomes similar to those for global-knockout *Pacc^–/–^* mice. Thus, knockout of *Pacc1* in leukocytes or other cell types is unlikely to exert significant indirect effects on the function of PAC in osteoclast fusion.

Attachment of TRAP^+^ mononuclear preosteoclasts to the bone surface initiates fusion to form polarized, multinucleated osteoclasts (44). The compartmentalized resorption environment is established by a circumferential attachment sealing zone (45). The plasma membrane within the sealing zone develops a ruffled border with abundant V-type H1-ATPase proton pump activity (46). Across this membrane, the osteoclasts actively secrete HCl into the compartment to dissolve the bone matrix. First, the ATPase proton pump inserts H^+^ into the resorption compartment, and then chloride ions passively cross the membrane via the chloride channel (21). These 2 steps involving the proton pump and chloride channel form HCl to acidify the resorption compartment and alkalinize the cytoplasm. Under the physiological condition of bone remodeling, the basolateral bicarbonate chloride exchanger corrects the cytoplasmic alkalinization by compensating for cytoplasmic chloride loss (47, 48), while the PAC channel is involved in chloride exchange on cell membrane as well. However, at low pH in the porous endplates, PAC is highly activated to promote chloride transport, which leads to aberrant osteoclast fusion and the development of LBP (Figure 7E). The aberrant osteoclast activity leads to the secretion of many factors, including netrin-1 and PDGF-BB, to induce sensory innervation and angiogenesis in the porous endplates that ultimately lead to LBP and spine degeneration (5, 37). Knockout of *Pacc1* substantially reduced endplate porosity and LBP. Therefore, PAC expression and activation in osteoclasts could be a potential therapeutic target for LBP or joint arthritis pain.

## Methods

### Sex as a biological variable.

Our study exclusively examined male mice, as in our previous studies. It is unknown whether the findings are relevant for female mice.

### Mice and in vivo treatment.

*Pacc1^fl/fl^*, *Pacc1^–/–^*, and *Pacc1^+/+^* mice were generated as previously described (10). The TRAP-Cre mouse strain was obtained from J.J. Windle (Virginia Commonwealth University, Richmond, Virgina, USA). Heterozygous TRAP-Cre mice were crossed with *Pacc1^fl/fl^* mice. The offspring were intercrossed to generate the following genotypes: WT, TRAP-Cre (mice expressing Cre recombinase driven by the *Trap* promoter), *Pacc1^fl/fl^* (mice homozygous for the *Pacc1* flox allele, referred to herein as *Pacc1^WT^*), and TRAP-Cre *Pacc1^fl/fl^* (conditional deletion of *Pacc1* in TRAP lineage cells, referred to herein as *Pacc1_TRAP_^−/−^*). The genotypes were determined by PCR analyses of genomic DNA, which was extracted from mouse tails with the following primers: WT/KO genotyping for *Pacc1* forward, 5′-TCCTGTTTGGACTCGGAACT-3′, reverse, 5′-TGGTAGCTGTGCCTGATGTC-3′; TMEM206_REV1, 5′-TCCTCACATAAGGGGCATG-3′; TRAP-Cre forward, 5′-ATATCTCACGTACTGACGGTGGG-3′, reverse, 5′-CTGTTTCACTATCCAGGTTACGG-3′; and *Pacc1* loxP allele forward, 5′-GAAGCCAGGCCATTCTTTTT-3′, reverse, 5′- GCTCAAGGAAACCACTGAGG -3′.

We performed LSI surgery on 2-month-old male mice that were WT, *Pacc1^–/–^*, *Pacc1^WT^*, or *Pacc1^fl/fl^*. Briefly, the mice were anesthetized with ketamine (100 mg/kg, i.p.) and xylazine (10 mg/kg, i.p.). Then, the LSI mouse model was created by resecting the L3–L5 spinous processes and the supraspinous and interspinous ligaments to induce LSI (*n* = 10–12 mice per group). Sham operations involved detachment of the posterior paravertebral muscles from L3–L5 only in a separate group of mice (*n* = 10–12 mice per group) (49). All mice were maintained at the animal facility of The Johns Hopkins University School of Medicine.

### Human tissue samples.

Human joint tissue samples were procured with IRB approval.

### Behavioral testing.

Behavioral testing was performed using *Pacc1^–/–^* and *Pacc1^WT^* mice that had undergone sham or LSI surgery. All behavioral tests were conducted by the same blinded investigator in the study group. Pressure thresholds were measure (SMALGO algometer, Bioseb) as pressure hyperalgesia (50). The L4–L5 spine was pressed by a 5 mm diameter sensor tip while the mice were gently restrained. The pressure force was gradually increased from 0 at a speed of 50 g/s until an audible vocalization was heard. The pressure force was read using BIO-CIS software (Bioseb). A cutoff force was set at 500 g to prevent tissue trauma. The mice were allowed to rest for 15 minutes between tests, and the mean value was calculated as the pressure tolerance threshold.

For the Von Frey test, we used a 0.4 g Von Frey filament (Stoelting) to determine the hind PWF. Mice were placed on a wire metal mesh grid covered with a black plastic cage. Mice were allowed to acclimatize to the environment for at least 30 minutes before testing. The mid-plantar surface was stimulated by the filament for 2 seconds. The PWF was recorded as the result of mechanical nociceptive threshold of the mice in response to 10 applications.

Spontaneous wheel-running activity was recorded using activity wheels designed for mice (model BIO-ACTIVW-M, Bioseb). The software enabled recording of the activities of a mouse in the wheel cage. The mice were acclimatized to the environment overnight before testing. The test lasted for 48 hours for each mouse. The parameters of the spontaneous activity were automatically recorded.

### μCT.

Mice were euthanized by isoflurane and perfused with 10% buffered formalin. For the analysis of endplates, the L3–L5 lumbar spine was collected and examined by μCT (voltage, 55 kVp; current, 181 μA; 9.0 μm per pixel) (Skyscan, 1172). For the analysis of femurs, the femur was collected and examined by μCT (voltage, 65 kVp; current, 153 μA; 9.0 μm per pixel) (Skyscan, 1172). Images were reconstructed using NRecon, version 1.6, software (Skyscan). Quantitative analysis of the μCT results was performed using CTAn, version 1.9, software (Skyscan). For the endplates, 6 consecutive images of the L4–L5 caudal endplates and L5 vertebrae (coronal view) were selected to show the 3D reconstruction results using CTVol, version 2.0, software (Skyscan). For the femurs, cross-sectional images were created of the femur for 3D analyses of trabecular bone using CTVol, version 2.0, software (Skyscan).

### Histochemistry, immunofluorescence, and histomorphometry for histological sections.

At the time of euthanasia, the L3–L5 lumbar spine and femur samples were collected and fixed in 10% buffered formalin for 24 hours. Both human and mouse bone samples were decalcified at 4°C using 0.5 M ethylenediamine tetra acetic acid for 2 months or 3 weeks with constant shaking and then embedded in paraffin or 8% gelatin in the presence of 20% sucrose and 2% polyvinylpyrrolidone. Coronal-oriented sections (4 μm thick) of the L4–L5 lumbar spine were processed for SOFG and TRAP (MilliporeSigma) staining using a standard protocol. Sections of human tibia tissue (4 μm thick) were used for coimmunofluorescence staining of PAC and TRAP. Coronal-oriented sections (40 μm thick) were prepared for sensory nerve–related immunofluorescence staining, and 10 μm thick coronal-oriented sections were used for other immunofluorescence staining using a standard protocol. The sections were incubated with primary antibodies against CGRP (1:100; ab81887, Abcam), PAC (1:500; noncommercial antibody), and TRAP (1:200; ab191406, Abcam) for 48 hours at 4°C. Then, the corresponding secondary antibodies were added onto the sections for 1 hour while avoiding light. The sections were then counterstained with DAPI (Vector Laboratories, H-1200). The sample images were observed and captured by a fluorescence microscope (Olympus BX51, DP71) or confocal microscope (Zeiss LSM 780). ImageJ (NIH) software was used for quantitative analysis.

### Cell isolation and culture.

The hind limbs of 8-week-old mice were harvested by carefully removing the attached soft tissue. Bone marrow cells were collected by cutting both ends of the tibia and femur and then flushing the marrow with a syringe using α–minimum essential medium (α-MEM) (MilliporeSigma). Whole bone marrow cells were collected following centrifugation for 15 minutes at 1,000 rpm and then cultured in α-MEM with 10% FBS (MilliporeSigma) at 37°C in a 5% CO_2_-humidified incubator. After 24 hours, the nonadherent cells floating in the culture media were collected and cultured in α-MEM with M-CSF (30 ng/mL). After 3 days, the macrophage lineage cells were collected by digesting the adherent cells with Versene Solution (Thermo Fisher Scientific). The BMMs were reseeded in 6-well plates (5 × 10^5^ cells per well), 24-well plates with coverslips (5 × 10^5^ cells per well), or 96-well plates with bone slices (1 × 10^6^ cells per well) and cultured in α-MEM containing 30 ng/mL M-CSF and 100 ng/mL RANKL (PeproTech). The pH level of the cell culture medium was calibrated using a blood gas analyzer at the Johns Hopkins Medical Laboratory.

### RT-PCR.

Total RNA was extracted from the cells using TRIzol reagent (Invitrogen, Thermo Fisher Scientific) according to the manufacturer’s instructions. The purity of RNA was tested by measuring the ratio of absorbance at 260 nm over 280 nm. For RT-PCR, 1 μg RNA was reverse transcribed into cDNA using the SuperScript First-Strand Synthesis System (Invitrogen, Thermo Fisher Scientific), and then RT-PCR was performed with SYBR Green Master Mix (QIAGEN) on a C1000 Thermal Cycler (Bio-Rad Laboratories). Relative expression was calculated for each gene by the 2^–ΔΔ^ Ct method, with glyceraldehyde 3-phosphate dehydrogenase for normalization. The following primers were used for RT-PCR: *Pacc1* forward, 5′-ATGATCCGACAAGAACTCTCCA-3′, reverse, 5′-AGCAGGACCGAGAAGACATTC-3′; *GAPDH* forward, 5′-AATGTGTCCGTCGTGGATCTGA-3′, reverse, 5′-AGTGTAGCCCAAGATGCCCTTC-3′; *St3gal1* forward, 5′-CCACAACGCTCTGATGGAGG-3′, reverse, 5′-AACAGTTCCTTGACGGTGTCG 3′; and *OC-STAMP* forward, 5′- CTGTAACGAACTACTGACCCAGC-3′, reverse 5′-CCAGGCTTAGGAAGACGAAGA-3′.

### Western blot analysis.

The cell lysates were centrifuged and separated by 10% SDS-PAGE and transferred onto a polyvinylidene difluoride membrane (Bio-Rad Laboratories). After blocking with 5% BSA in Tris-buffered saline containing 0.05% Tween-20 (TBST), the membrane was incubated with specific primary antibodies at 4°C overnight. The membrane was then washed with TBST and incubated with HRP-conjugated secondary antibodies. Protein was detected using an ECL kit (Thermo Fisher Scientific). Primary antibodies recognizing mouse PAC (1:500; Ab99055, Abcam), NFATc1 (1:500; MA3-024, Thermo Fisher Scientific), St3gal1 (1:1,000; LS-C185763-100, Lifespan), β-actin (1:1,000; 3700, Cell Signaling Technology), and β-tubulin (1:1,000; Ab108342, Abcam) were used to determine the protein concentrations in the lysates.

### ChIP assay.

Cells were added with formaldehyde to crosslink proteins to DNA, and cells were lysed in 1.5 mL lysis buffer (50 mM HEPES, pH 7.5, 140 mM NaCl, 1 mM EDTA, 1% Triton X-100, 0.1% sodium deoxy cholate, 0.1% sodium dodecyl sulfate). Cell lysates were sonicated at 2 seconds on/15 seconds off for 3 rounds using a Bioruptor ultrasonic cell disruptor (Diagenode) to shear genomic DNA to a mean fragment size of 150–250 bp. Of the sample, 1% was removed for use as the input control. ChIP was performed according to the protocol provided with the Simple Chip Enzymatic Chromatin IP Kit (Cell Signaling Technology) using antibodies against NFATc1 (Thermo Fisher Scientific). Anti-RNA polymerase II and control IgG were used as positive and negative controls, respectively. After washing and de-crosslinking, the precipitated DNA was purified using a QIAquick PCR Purification kit (QIAGEN). The following PCR primers were used to detect the NFATc1 binding site: site 1, forward, 5′-ACTTGCTTTCCTGCTCCT-3′, reverse, 5′-TTCCCTGTCTATCTTCTTTCTA-3′; site 2, forward, 5′-GCTAACCTGGACGCTTGT-3′, reverse, 5′-TTTGTTTGTGCTTGCTCT-3′; site 3, forward, 5′-GGCTGATATTGGTTTGTA-3′, reverse, 5′-GTCCCTTCTTGTTTGTCT -3′.

### Whole-cell electrophysiology.

For whole-cell patch-clamp recordings on the proton-activated Cl^–^ channel, the extracellular solution contained 145 mM NaCl, 2 mM KCl, 2 mM MgCl_2_, 1.5 mM CaCl_2_, 10 mM HEPES, and 10 mM glucose (300 mOsm/kg; pH 7.3 with NaOH). Different acidic pH solutions were made of the same ionic composition without HEPES but with 5 mM Na3-citrate as the buffer, and the pH was adjusted using citric acid. Patch pipettes were fabricated from borosilicate glass (Sutter Instruments) and pulled with a Model P-1000 multistep puller (Sutter Instruments) and had a resistance of 2–4 MΩ when filled with an internal solution containing 135 mM CsCl, 1 mM MgCl_2_, 2 mM CaCl_2_, 10 mM HEPES, 5 mM EGTA, and 4 mM MgATP (280–290 mOsm/kg; pH 7.2 with CsOH). Extracellular solutions were applied using a gravity perfusion system with a small tip that was approximately 100–200 μm away from the recording cell. All experiments were done at 37°C. Recordings were made with a MultiClamp 700B amplifier and a 1550B digitizer (Molecular Devices). Signals were filtered at 2 kHz and digitized at 10 kHz. The capacitive transients were compensated just before each measurement, and the series resistance was then routinely compensated for by at least 80%.

For PAC current recording, cells were held at 0 mV, and voltage ramps (500 ms duration) were applied from –100 to +100 mV. Data were analyzed using Clampfit 10.6 and GraphPad Prism 6 software (GraphPad Software).

### Bone resorption assay.

To assess the effect of PAC-induced bone resorption in acidic condition, an osteoclast bone resorption assay was performed using a commercially available bone resorption assay kit (Cosmo Bio). Briefly, BMMs isolated from *Pacc1^–/–^* and *Pacc1^+/+^* mice were seeded on bone slices in 24-well plates and cultured in acidic or neutral osteoclastogenic medium for 7 days. The resorption pits on the hydroxyapatite surface were imaged under a microscope.

### Staining of the osteoclasts.

Cells were cultured in osteoclastogenic medium for 1, 3, or 5 days, and fixed for 10 minutes with 4% paraformaldehyde. A TRAP staining kit (MilliporeSigma) was used to detect TRAP^+^ cells according to the manufacturer’s instructions. Fluorescence staining of F-actin with phalloidin was used to observe action ring formation. Sample images were captured with a fluorescence microscope (Olympus BX51, DP71).

### siRNA interference.

For in vitro siRNA interference, mouse *St3gal1* siRNA (150809, Thermo Fisher Scientific) and control siRNA (4390843, Thermo Fisher Scientific) were transfected into primary *Pacc1^+/+^* and *Pacc1^–/–^* cells using a Lipofectamine RNAiMAX transfection kit (13778030, Thermo Fisher Scientific) and Opti-MEM Reduced Serum Medium (31985062, Thermo Fisher Scientific) according to the manufacturer’s guidelines.

### Endplate pH measurement.

To conclusively determine the presence of acidic conditions in the spinal endplate area, a Micro combination pH electrode with a needle tip was used for accurate in vivo pH assessments (9863BN Micro pH Electrode, Thermo Fisher Scientific). Both LSI and sham-operated mice were sedated via i.p. injections of ketamine (100 mg/kg) and xylazine (10 mg/kg). The L3–L5 endplates were then surgically exposed. To protect the surrounding tissues and precisely target the area of interest, sterile gauze was applied. For each mouse, the average pH values at the L3, L4, and L5 endplates were calculated, ensuring accurate representation of the local pH environment within the spinal endplate region.

### Statistics.

All data analyses were performed using SPSS, version 24.0, software (IBM). Data are presented as the mean ± SD. In general, an independent sample, 2-tailed *t* test was used for comparisons among 2 groups, and 1-way ANOVA with Bonferroni’s post hoc test was used for comparisons among multiple groups. For in vivo studies, 2-way, repeated-measures ANOVA with Bonferroni’s post hoc test was performed to test the effect of LSI surgery or genotype on behavior test results at different time points. The effects of LSI surgery or genotype on endplate porosity, osteoclast function, and gene expression were analyzed by 2-way ANOVA with Bonferroni’s post hoc test. For in vitro studies, 2-way ANOVA with Bonferroni’s post hoc test was performed to test the effect of acidic condition or genotype on osteoclast differentiation and function. For all experiments, a *P* value of less than 0.05 was considered significant. All inclusion/exclusion criteria were preestablished, and no samples or animals were excluded from the analysis. No statistical method was used to predetermine the sample size. The experiments were randomized, and the investigators were blinded to allocation during experiments and outcome assessment.

### Study approval.

The IRB of Johns Hopkins University School of Medical granted a waiver of consent for human participants, as the specimens comprised deidentified tissue archived by the pathology department. This approach aligned with the FDA’s regulations on consent waiver (Organization Policy FDA 50.1), permitting the use of such samples for research without individual consent due to the anonymity and preexisting status of the tissue.

### Data availability.

Values for all data points in graphs are reported in the Supporting Data Values file.

## Author contributions

XC, ZQ, PX, and WZ conceived of the study. PX, SW, JC, and MW designed and conducted primary experiments. XC and PX wrote the manuscript. XC, WZ, and JZ contributed to crafting the rebuttal and revising the manuscript. WZ and JY contributed to the experiments for the revision, and MS helped with experiments. Mouse anti–human PAC monoclonal antibodies were provided by ZQ.

## Supplementary Material

Supplemental data

Supporting data values

## Version 1 08/28/2024

In-Press Preview

## Version 2 10/15/2024

Electronic publication
