# Supplementary material for: Proton-activated chloride channel increases endplate porosity and pain in a mouse spine degeneration model
Source: J Clin Invest. 2024 Aug 28;134(20):e168155. doi: 10.1172/JCI168155 (PMC11473161; doi:10.1172/JCI168155)
Supplement: Supplemental data [file jci-134-168155-s066.pdf]

Supplemental Figures and legends:

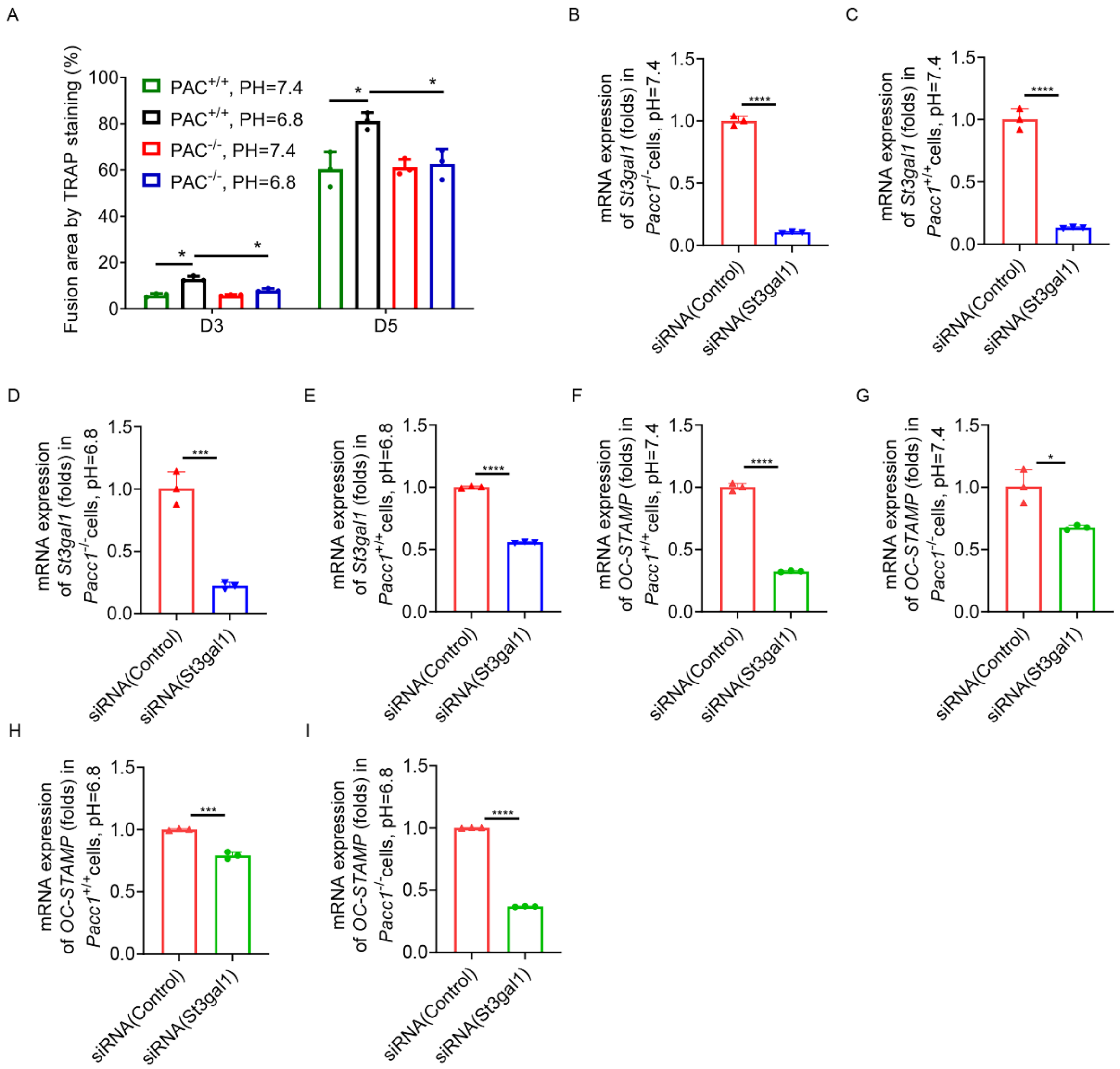

Supplemental Figure 1. RT-PCR analysis of the expression of osteoclast fusion related gene.

(A) Quantitative analysis of TRAP staining by fusion area for cells isolated from *Pacc1*$^{+/+}$ or *Pacc1*$^{-/-}$ mice cultured in neural or acidic medium at day 3 and day 5. (B-E) The quantitative analysis of the gene expression of *St3gal1* in *St3gal1* siRNA treated osteoclasts compared to control siRNA treated in *Pacc1*$^{-/-}$ pH 7.4 (B), *Pacc1*$^{+/+}$ pH 7.4 (C), *Pacc1*$^{-/-}$ pH 6.8 (D), *Pacc1*$^{+/+}$ pH 6.8 (E). (F-I) The quantitative analysis of the gene expression of *OC-STAMP* in *St3gal1* siRNA treated osteoclasts compared to control siRNA treated in *Pacc1*$^{+/+}$ pH 7.4 (F), *Pacc1*$^{-/-}$ pH 7.4 (G), *Pacc1*$^{+/+}$ pH 6.8 (H), *Pacc1*$^{-/-}$ pH 6.8 (I), *$p < 0.05$, ***$p < 0.005$, ****$p < 0.001$.

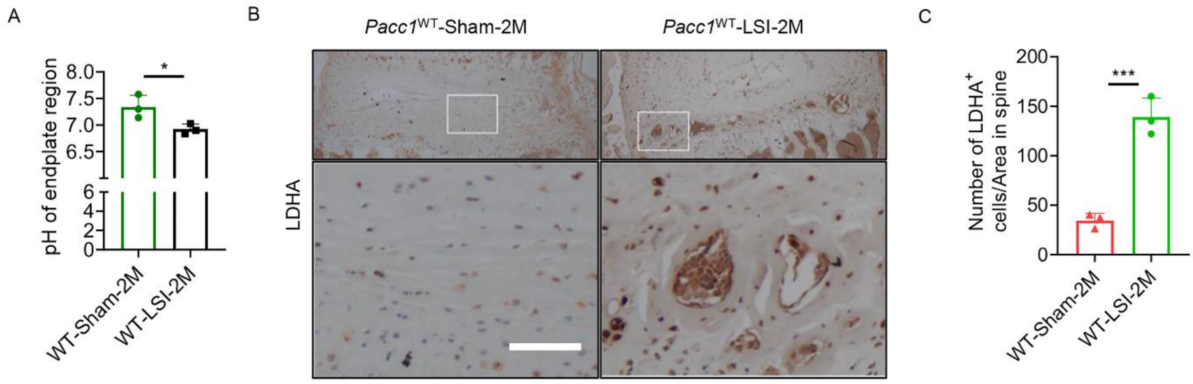

Supplemental Figure 2. IHC staining of LDHA in spine tissue section.

(A) The quantitative results of the average pH value at L3-L5 endplates in WT sham or LSI mice. (B) The representative images of IHC staining of LDHA in the spine section in *Pacc1*^{WT} mice with sham or LSI surgery for two months. Scale bar: 0.5 mm. (C) The quantitative analysis of the number of LDHA positive cells per area, *$p < 0.05$, ***$p < 0.005$.
